# Crystal structure of 2,6-bis­(2-hy­droxy-5-methyl­phen­yl)-4-phenyl­pyridinium bromide di­chloro­methane hemisolvate hemihydrate

**DOI:** 10.1107/S2056989015021386

**Published:** 2015-11-18

**Authors:** Badma N. Mankaev, Kirill V. Zaitsev, Sergey S. Karlov, Mikhail P. Egorov, Andrei V. Churakov

**Affiliations:** a N.D. Zelinsky Institute of Organic Chemistry, Leninsky prospekt 47, Moscow 119991, Russian Federation; b Department of Chemistry, M.V. Lomonosov Moscow State University, Moscow, Russian Federation; c Institute of General and Inorganic Chemistry, Russian Academy of Sciences, Moscow, Russian Federation

**Keywords:** crystal structure, ONO-type ligands, pseudosymmetry, hydrogen bonding, π–π stacking

## Abstract

The asymmetric unit in the structure of the title compound, C_25_H_22_NO_2_^+^·Br ^−^·0.5CH_2_Cl_2_·0.5H_2_O, comprises two pseudosymmetry-related cations, two bromide anions, a di­chloro­methane molecule and a water mol­ecule of solvation. The two independent cations are conformationally similar with the comparative dihedral angles between the central pyridine ring and the three benzene substituent rings being 3.0 (2), 36.4 (1) and 24.2 (1)°, and 3.7 (2), 36.5 (1) and 24.8 (1)°, respectively. In the crystal, the cations, anions and water mol­ecules are linked through O—H⋯O and O—H⋯Br hydrogen bonds, forming an insular unit. Within the cations there are also intra­molecular N—H⋯O hydrogen bonds. Adjacent centrosymmetrically related aggregates are linked by π–π stacking inter­actions between the pyridine ring and a benzene ring in both cations [ring-centroid separations = 3.525 (3) and 3.668 (3) Å], forming chains extending across the *ac* diagonal. Voids between these chains are filled by dichloromethane molecules.

## Related literature   

For general background to the chemistry affording 2,6-bis-(2-hy­droxy­phen­yl)pyridines, see: Huang *et al.* (2012 ▸, 2013 ▸); Kire­enko *et al.* (2013 ▸); Klein *et al.* (2010 ▸); Li *et al.* (2000 ▸); Steinhauser *et al.* (2004 ▸); Zhang *et al.* (2006 ▸). For the closely related structure of the parent derivative compound, see: Silva *et al.* (1997 ▸). 
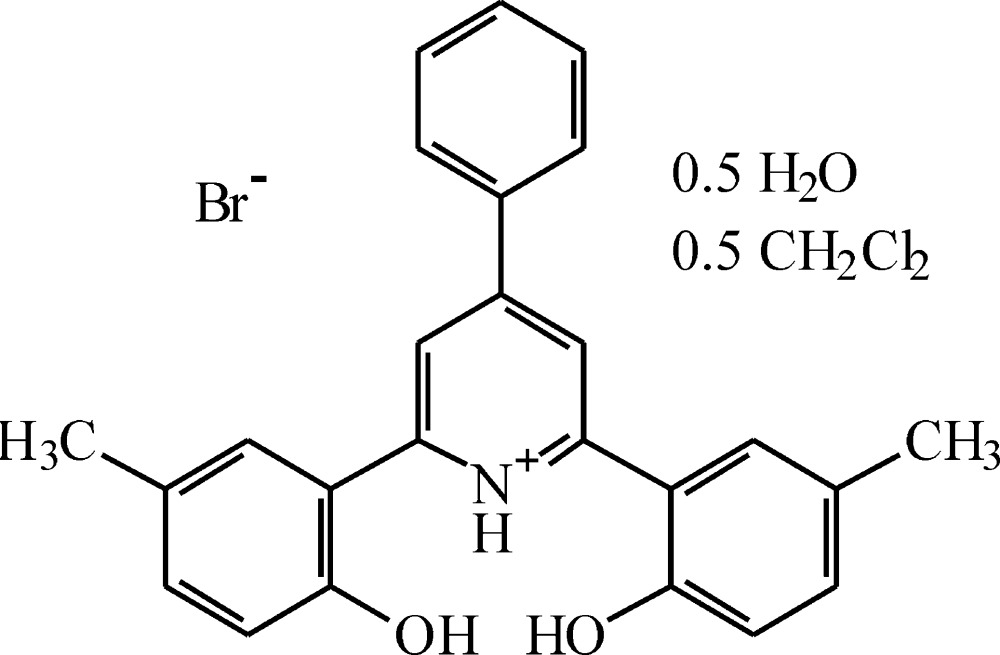


## Experimental   

### Crystal data  

2C_25_H_22_NO_2_^+^·2Br^−^·CH_2_Cl_2_·H_2_O
*M* *_r_* = 999.63
Monoclinic, 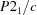

*a* = 14.7890 (12) Å
*b* = 17.5387 (14) Å
*c* = 19.0163 (15) Å
β = 112.577 (1)°
*V* = 4554.4 (6) Å^3^
*Z* = 4
Mo *K*α radiation
μ = 1.95 mm^−1^
*T* = 150 K
0.25 × 0.20 × 0.10 mm

### Data collection  

Bruker SMART APEXII diffractometer
Absorption correction: multi-scan (*SADABS*; Bruker, 2008 ▸) *T*_min_ = 0.642, *T*_max_ = 0.829
34435 measured reflections
8482 independent reflections
6350 reflections with *I* > 2σ(*I*)
*R*_int_ = 0.050

### Refinement  

*R*[*F* ^2^ > 2σ(*F* ^2^)] = 0.051
*wR*(*F* ^2^) = 0.132
*S* = 1.06
8482 reflections
585 parameters
15 restraints
H atoms treated by a mixture of independent and constrained refinement
Δρ_max_ = 0.99 e Å^−3^
Δρ_min_ = −0.92 e Å^−3^

### 

Data collection: *APEX2* (Bruker, 2008 ▸); cell refinement: *SAINT* (Bruker, 2008 ▸); data reduction: *SAINT*; program(s) used to solve structure: *SHELXS97* (Sheldrick, 2008 ▸); program(s) used to refine structure: *SHELXL97* (Sheldrick, 2008 ▸); molecular graphics: *SHELXTL* (Sheldrick, 2008 ▸); software used to prepare material for publication: *SHELXTL*.

## Supplementary Material

Crystal structure: contains datablock(s) I. DOI: 10.1107/S2056989015021386/zs2352sup1.cif

Structure factors: contains datablock(s) I. DOI: 10.1107/S2056989015021386/zs2352Isup2.hkl

Supporting information file. DOI: 10.1107/S2056989015021386/zs2352Isup3.mol

Supporting information file. DOI: 10.1107/S2056989015021386/zs2352Isup4.cml

**Fig. 1.**
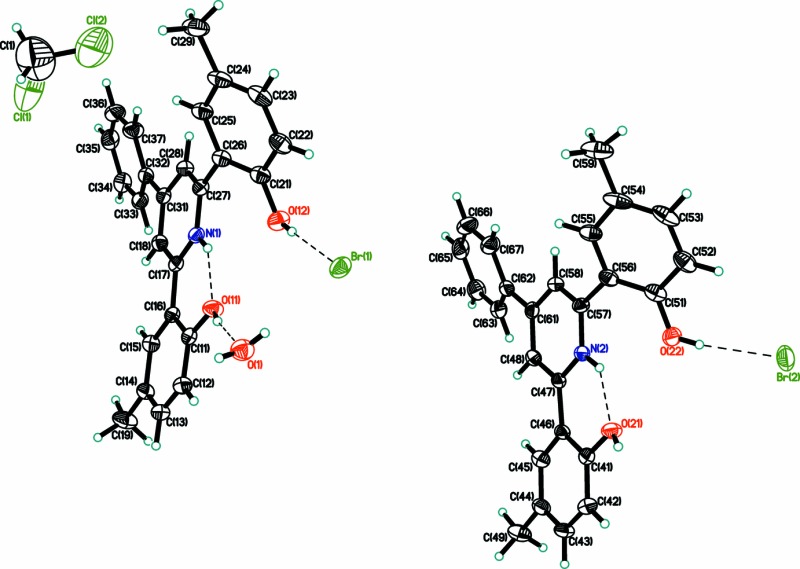
The asymmetric unit in the structure of the title compound, with displacement ellipsoids shown at the 50% probability level. Hydrogen bonds are shown as dashed lines.

. DOI: 10.1107/S2056989015021386/zs2352fig1.tif
The asymmetric unit in the structure of the title compound, with displacement ellipsoids shown at the 50% probability level. Hydrogen bonds are shown as dashed lines.

**Fig. 2.**
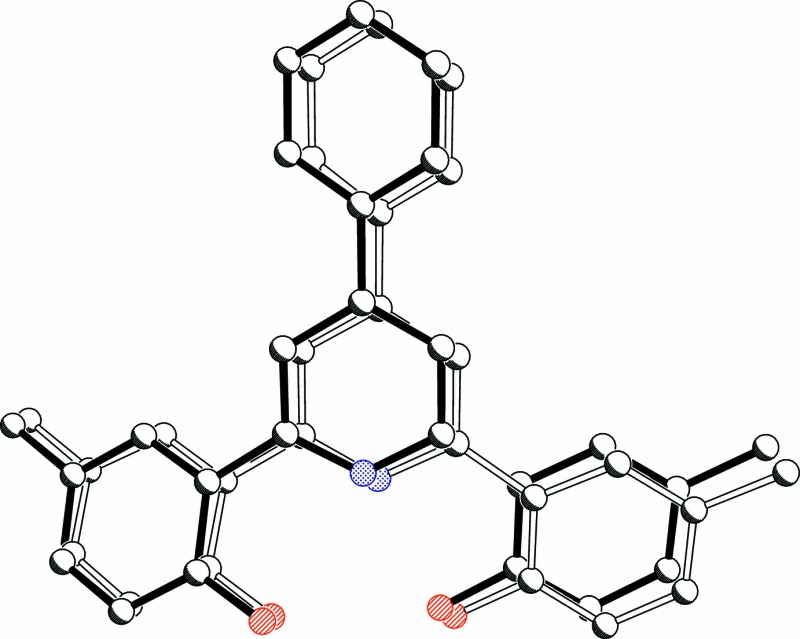
The result of superposition of one independent cation with another shifted by an x + 1/2, y + 1/2, z operation.

Click here for additional data file.x y z . DOI: 10.1107/S2056989015021386/zs2352fig2.tif
The result of superposition of one independent cation with another shifted by an *x* + 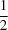, *y* + 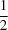, *z* operation.

**Fig. 3.**
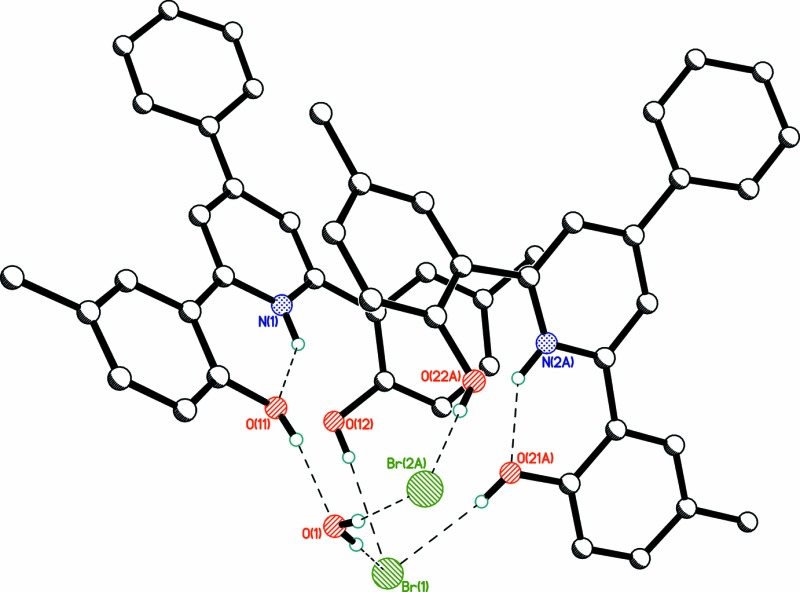
Insular hydrogen bonded aggregates in the structure. Hydrogen bonds are shown as dashed lines. Suffix A indicates the symmetry operator -x + 1, y - 1/2, -z + 1/2.

Click here for additional data file.A x y z . DOI: 10.1107/S2056989015021386/zs2352fig3.tif
Insular hydrogen bonded aggregates in the structure. Hydrogen bonds are shown as dashed lines. Suffix *A* indicates the symmetry operator −*x* + 1, *y* − 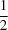, −*z* + 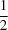.

**Fig. 4.**
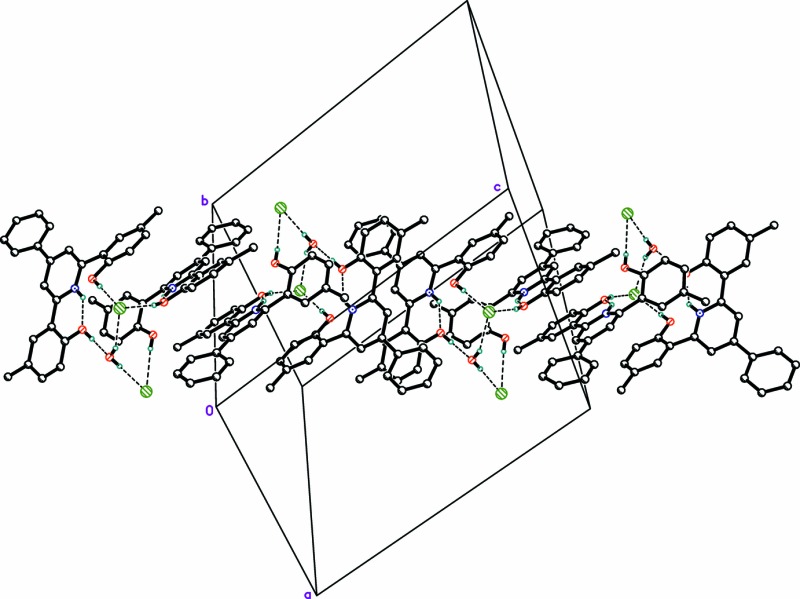
Chains formed by π–π stacking interactions between aromatic ring systems in adjacent H-bonded frameworks.

. DOI: 10.1107/S2056989015021386/zs2352fig4.tif
Chains formed by π–π stacking inter­actions between aromatic ring systems in adjacent H-bonded frameworks.

CCDC reference: 1436252

Additional supporting information:  crystallographic information; 3D view; checkCIF report

## Figures and Tables

**Table 1:** Hydrogen-bond geometry (Å, °)

*D*—H⋯*A*	*D*—H	H⋯*A*	*D*⋯*A*	*D*—H⋯*A*
N1—H1⋯O11	0.88	1.82	2.547 (4)	138
N2—H2⋯O21	0.88	1.85	2.575 (4)	138
O11—H3⋯O1	0.79 (2)	1.80 (3)	2.578 (4)	167 (5)
O12—H4⋯Br1	0.79 (2)	2.43 (2)	3.219 (3)	175 (5)
O21—H5⋯Br1^i^	0.79 (2)	2.41 (2)	3.200 (3)	170 (5)
O22—H6⋯Br2	0.79 (2)	2.35 (2)	3.131 (3)	170 (5)
O1—H7⋯Br2^ii^	0.80 (2)	2.41 (2)	3.206 (3)	173 (5)
O1—H8⋯Br1	0.79 (2)	2.61 (3)	3.365 (3)	159 (5)

Symmetry codes: (i) 
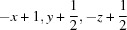
; (ii) 
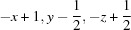
.

## supplementary crystallographic information

### S1. Comment

Interest in dianionic tridentate *ONO*-type ligands has grown steadily over the past several decades due to their ability to stabilize unusual metal oxidation states and the coordination geometry around metal centers. As a part of our investigation on the synthesis of tridentate ligands (Kireenko *et al.*, 2013; Huang *et al.*, 2013, 2012) we obtained and studied the structure of the title compound, 2(C_25_H_22_NO_2_^+^) . 2Br ^-^ . CH_2_Cl_2_ . H_2_O, which may be regarded as a precursor of a promising ligand for the preparation of complexes of main group metal elements.

The asymmetric unit comprises two independent ligand cations, two bromide anions, a dichlormethane and a water molecule of solvation (Fig. 1). The cations are related by pseudotranslation (one half of the *ab* diagonal) and possess very similar geometrical parameters and conformations. The comparative dihedral angles between the central pyridine ring and the three benzene substituent rings are 3.0 (2), 36.4 (1), 24.2 (1)° and 3.7 (2), 36.5 (1), 24.8 (1)°, respectively for cations 1 and 2. Figure 2 represents the superposition of one independent cation with another shifted by *x* + 0.5, *y* + 0.5, *z*. However, the bromide anions and the solvent water molecules do not satisfy this pseudosymmetry law.

In the crystal, the two crystallographically independent organic cations, the two bromide anions, and the water molecules are associated through moderately strong inter-species O—H···O and O—H···Br hydrogen bonds (Table 1), forming an insular framework (Fig. 3). Within the unit there are also intramolecular N—H···O hydrogen bonds. Adjacent centrosymmetrically- related aggregates are linked by π–π stacking interactions between the pyridine ring (N1–C31 in cation 1 and N2–C61 in cation 2) and a benzene ring (C11–C16 in cation 1 and C41–C46 in cation 2), giving ring centroid separations of 3.525 (3) and 3.668 (3) Å, respectively. This results in the formation of chains extending across the *ac* diagonal (Fig. 4).

### S2. Experimental

The precursor of the title salt, 2,6-bis(2'-hydroxy-5'-methylphenyl)-4- phenylpyridine, was obtained from 2-hydroxy-5-methylacetophenone *via* two parallel reactions: (*a*), condensation of the above acetophenone with benzaldehyde in the presence of NaOH and (*b*), iodination of above acetophenone in the presence of pyridine. The reaction of an equimolar mixture of the above intermediates with ammonium acetate led to formation of the precursor, with moderate yield.

NMR spectra of 2,6-bis(2'-hydroxy-5'-methylphenyl)-4-phenylpyridine: ^1^H NMR (CDCl_3_): δ 9.83 (s, 2H, OH), 7.86 (s, 2H, aromatic H atoms), 7.74 (d, J = 7.6 Hz, 2H, aromatic H atoms), 7.48–7.57 (m, 5H, aromatic H atoms), 7.15 (d, J = 8.3 Hz, 2H, aromatic H atoms), 6.98 (d, J = 8.3 Hz, 2H, aromatic H atoms), 2.37 (s, 6H, Me) p.p.m.. ^13^C NMR (CDCl_3_): δ 156.52, 154.43, 151.93, 138.15, 132.17, 129.53, 129.20, 129.02, 128.29, 127.20, 121.36, 117.88, 117.76 (aromatic carbons), 20.60 (Me) p.p.m.

Crystals of the title compound suitable for X-ray analysis were precipitated from the reaction of 2,6-bis(2'-hydroxy-5'-methylphenyl)-4-phenylpyridine with silicon tetrabromide in dichloromethane.

### S3. Refinement

All hydrogen atoms on aromatic atoms (both C and N) and methyl groups were placed in calculated positions and refined using a riding model, with C—H = 0.95–0.98 Å, with N—H = 0.88 Å, and with *U*_iso_(H) = 1.2 *U*_eq_(C,N) or 1.5 *U*_eq_(C) for methyl H atoms. A rotating model was applied to the methyl groups. All hydroxy and water hydrogen atoms were found from difference Fourier syntheses and refined with *U*_iso_(H) = 1.5 *U*_eq_(O) and restrained O—H distances (SADI). Three outliers (-1 1 1, 0 1 1, 1 1 0) were omitted from the data set in the last cycles of refinement.

### Crystal data

**Table:**

2C_25_H_22_NO_2_^+^·2Br^−^·CH_2_Cl_2_·H_2_O	*F*(000) = 2048
*M**_r_* = 999.63	*D*_x_ = 1.458 Mg m^−^^3^
Monoclinic, *P*2_1_/*c*	Mo *K*α radiation, λ = 0.71073 Å
Hall symbol: -P 2ybc	Cell parameters from 6112 reflections
*a* = 14.7890 (12) Å	θ = 2.2–23.3°
*b* = 17.5387 (14) Å	µ = 1.95 mm^−^^1^
*c* = 19.0163 (15) Å	*T* = 150 K
β = 112.577 (1)°	Prism, orange
*V* = 4554.4 (6) Å^3^	0.25 × 0.20 × 0.10 mm
*Z* = 4	

### Data collection

**Table:**

Bruker SMART APEXII diffractometer	8482 independent reflections
Radiation source: fine-focus sealed tube	6350 reflections with *I* > 2σ(*I*)
Graphite monochromator	*R*_int_ = 0.050
ω scans	θ_max_ = 25.5°, θ_min_ = 1.5°
Absorption correction: multi-scan (*SADABS*; Bruker, 2008)	*h* = −17→17
*T*_min_ = 0.642, *T*_max_ = 0.829	*k* = −21→21
34435 measured reflections	*l* = −23→23

### Refinement

**Table:**

Refinement on *F*^2^	Primary atom site location: structure-invariant direct methods
Least-squares matrix: full	Secondary atom site location: difference Fourier map
*R*[*F*^2^ > 2σ(*F*^2^)] = 0.051	Hydrogen site location: mixed
*wR*(*F*^2^) = 0.132	H atoms treated by a mixture of independent and constrained refinement
*S* = 1.06	*w* = 1/[σ^2^(*F*_o_^2^) + (0.0499*P*)^2^ + 10.5059*P*] where *P* = (*F*_o_^2^ + 2*F*_c_^2^)/3
8482 reflections	(Δ/σ)_max_ = 0.001
585 parameters	Δρ_max_ = 0.99 e Å^−^^3^
15 restraints	Δρ_min_ = −0.92 e Å^−^^3^

### Special details

**Table:**

Geometry. All e.s.d.'s (except the e.s.d. in the dihedral angle between two l.s. planes) are estimated using the full covariance matrix. The cell e.s.d.'s are taken into account individually in the estimation of e.s.d.'s in distances, angles and torsion angles; correlations between e.s.d.'s in cell parameters are only used when they are defined by crystal symmetry. An approximate (isotropic) treatment of cell e.s.d.'s is used for estimating e.s.d.'s involving l.s. planes.
Refinement. Refinement of *F*^2^ against ALL reflections. The weighted *R*-factor *wR* and goodness of fit *S* are based on *F*^2^, conventional *R*-factors *R* are based on *F*, with *F* set to zero for negative *F*^2^. The threshold expression of *F*^2^ > σ(*F*^2^) is used only for calculating *R*-factors(gt) *etc*. and is not relevant to the choice of reflections for refinement. *R*-factors based on *F*^2^ are statistically about twice as large as those based on *F*, and *R*- factors based on ALL data will be even larger.

### Fractional atomic coordinates and isotropic or equivalent isotropic displacement parameters (Å^2^)

**Table:**

	*x*	*y*	*z*	*U*_iso_*/*U*_eq_	
Br1	0.37349 (4)	0.71568 (2)	0.20516 (2)	0.03429 (13)	
Br2	0.99858 (4)	1.19630 (3)	0.23428 (3)	0.04276 (15)	
O1	0.2325 (2)	0.71111 (18)	0.30809 (18)	0.0353 (7)	
H7	0.1742 (17)	0.711 (3)	0.295 (3)	0.053*	
H8	0.253 (4)	0.719 (3)	0.276 (2)	0.053*	
C1	0.2288 (7)	0.1114 (6)	0.2630 (6)	0.132 (4)	
H1A	0.2072	0.0637	0.2334	0.159*	
H1B	0.1698	0.1427	0.2546	0.159*	
Cl1	0.28379 (17)	0.08839 (13)	0.35994 (17)	0.1249 (9)	
Cl2	0.3025 (3)	0.15925 (15)	0.2295 (2)	0.1806 (16)	
N1	0.3903 (2)	0.45926 (17)	0.38647 (17)	0.0214 (7)	
H1	0.3658	0.5022	0.3628	0.026*	
O11	0.3172 (2)	0.59130 (16)	0.38313 (16)	0.0282 (6)	
H3	0.284 (3)	0.625 (2)	0.359 (3)	0.049 (16)*	
O12	0.4366 (2)	0.55415 (17)	0.29039 (16)	0.0326 (7)	
H4	0.418 (3)	0.5929 (18)	0.268 (2)	0.034 (14)*	
C11	0.3174 (3)	0.5801 (2)	0.4537 (2)	0.0233 (8)	
C12	0.2787 (3)	0.6347 (2)	0.4869 (2)	0.0288 (9)	
H12	0.2498	0.6795	0.4592	0.035*	
C13	0.2816 (3)	0.6247 (2)	0.5598 (2)	0.0306 (10)	
H13	0.2542	0.6625	0.5815	0.037*	
C14	0.3240 (3)	0.5603 (2)	0.6016 (2)	0.0281 (9)	
C15	0.3605 (3)	0.5048 (2)	0.5675 (2)	0.0259 (9)	
H15	0.3882	0.4599	0.5954	0.031*	
C16	0.3582 (3)	0.5126 (2)	0.4933 (2)	0.0218 (8)	
C17	0.3961 (3)	0.4507 (2)	0.4591 (2)	0.0217 (8)	
C18	0.4351 (3)	0.3826 (2)	0.4955 (2)	0.0223 (8)	
H18	0.4397	0.3743	0.5461	0.027*	
C19	0.3313 (4)	0.5514 (3)	0.6828 (3)	0.0428 (12)	
H19A	0.2658	0.5551	0.6841	0.064*	
H19B	0.3732	0.5917	0.7145	0.064*	
H19C	0.3597	0.5015	0.7025	0.064*	
C21	0.4112 (3)	0.4948 (2)	0.2400 (2)	0.0276 (9)	
C22	0.3957 (3)	0.5043 (3)	0.1632 (2)	0.0314 (10)	
H22	0.4036	0.5531	0.1448	0.038*	
C23	0.3689 (3)	0.4430 (3)	0.1142 (2)	0.0336 (10)	
H23	0.3575	0.4506	0.0620	0.040*	
C24	0.3583 (3)	0.3703 (3)	0.1392 (2)	0.0335 (10)	
C25	0.3768 (3)	0.3605 (2)	0.2163 (2)	0.0293 (9)	
H25	0.3717	0.3110	0.2345	0.035*	
C26	0.4027 (3)	0.4217 (2)	0.2674 (2)	0.0256 (9)	
C27	0.4198 (3)	0.4063 (2)	0.3481 (2)	0.0238 (8)	
C28	0.4607 (3)	0.3400 (2)	0.3845 (2)	0.0251 (9)	
H28	0.4844	0.3033	0.3590	0.030*	
C29	0.3259 (4)	0.3031 (3)	0.0851 (3)	0.0436 (12)	
H29A	0.3653	0.2582	0.1089	0.065*	
H29B	0.3347	0.3152	0.0379	0.065*	
H29C	0.2566	0.2923	0.0736	0.065*	
C31	0.4678 (3)	0.3259 (2)	0.4590 (2)	0.0237 (8)	
C32	0.5077 (3)	0.2525 (2)	0.4977 (2)	0.0255 (9)	
C33	0.5463 (3)	0.2452 (2)	0.5764 (2)	0.0280 (9)	
H33	0.5508	0.2891	0.6069	0.034*	
C34	0.5781 (3)	0.1763 (2)	0.6115 (3)	0.0330 (10)	
H34	0.6042	0.1730	0.6655	0.040*	
C35	0.5721 (3)	0.1118 (2)	0.5680 (3)	0.0365 (11)	
H35	0.5939	0.0640	0.5920	0.044*	
C36	0.5345 (4)	0.1173 (2)	0.4896 (3)	0.0394 (11)	
H36	0.5302	0.0731	0.4597	0.047*	
C37	0.5029 (3)	0.1869 (2)	0.4544 (3)	0.0344 (10)	
H37	0.4778	0.1902	0.4004	0.041*	
N2	0.8706 (2)	0.96667 (17)	0.38890 (17)	0.0207 (7)	
H2	0.8481	1.0100	0.3653	0.025*	
O21	0.8014 (2)	1.10189 (16)	0.38610 (16)	0.0292 (7)	
H5	0.763 (3)	1.134 (2)	0.365 (2)	0.040 (15)*	
O22	0.9207 (2)	1.05281 (16)	0.29143 (16)	0.0323 (7)	
H6	0.934 (3)	1.0917 (18)	0.277 (3)	0.040 (14)*	
C41	0.8155 (3)	1.0944 (2)	0.4612 (2)	0.0234 (8)	
C42	0.7898 (3)	1.1537 (2)	0.4984 (2)	0.0309 (10)	
H42	0.7609	1.1987	0.4712	0.037*	
C43	0.8060 (3)	1.1474 (2)	0.5745 (2)	0.0314 (10)	
H43	0.7870	1.1880	0.5990	0.038*	
C44	0.8495 (3)	1.0829 (2)	0.6162 (2)	0.0284 (9)	
C45	0.8743 (3)	1.0243 (2)	0.5782 (2)	0.0265 (9)	
H45	0.9038	0.9798	0.6061	0.032*	
C46	0.8581 (3)	1.0274 (2)	0.5005 (2)	0.0216 (8)	
C47	0.8837 (3)	0.9611 (2)	0.4636 (2)	0.0208 (8)	
C48	0.9198 (3)	0.8924 (2)	0.4998 (2)	0.0231 (8)	
H48	0.9288	0.8861	0.5517	0.028*	
C49	0.8700 (3)	1.0787 (3)	0.7002 (2)	0.0380 (11)	
H49A	0.8921	1.0271	0.7189	0.057*	
H49B	0.8101	1.0906	0.7085	0.057*	
H49C	0.9212	1.1155	0.7277	0.057*	
C51	0.8924 (3)	0.9936 (2)	0.2414 (2)	0.0273 (9)	
C52	0.8854 (3)	1.0006 (3)	0.1662 (2)	0.0331 (10)	
H52	0.9006	1.0477	0.1485	0.040*	
C53	0.8561 (3)	0.9384 (3)	0.1179 (2)	0.0357 (11)	
H53	0.8510	0.9438	0.0668	0.043*	
C54	0.8339 (3)	0.8682 (3)	0.1414 (2)	0.0352 (11)	
C55	0.8426 (3)	0.8619 (2)	0.2168 (2)	0.0271 (9)	
H55	0.8277	0.8146	0.2341	0.033*	
C56	0.8727 (3)	0.9230 (2)	0.2678 (2)	0.0246 (9)	
C57	0.8900 (3)	0.9097 (2)	0.3485 (2)	0.0224 (8)	
C58	0.9268 (3)	0.8424 (2)	0.3845 (2)	0.0220 (8)	
H58	0.9415	0.8023	0.3571	0.026*	
C59	0.8031 (4)	0.8003 (3)	0.0885 (3)	0.0470 (13)	
H59A	0.8494	0.7584	0.1096	0.071*	
H59B	0.8026	0.8146	0.0385	0.071*	
H59C	0.7373	0.7840	0.0829	0.071*	
C61	0.9431 (3)	0.8322 (2)	0.4614 (2)	0.0225 (8)	
C62	0.9826 (3)	0.7596 (2)	0.5007 (2)	0.0256 (9)	
C63	1.0329 (3)	0.7553 (2)	0.5791 (2)	0.0297 (9)	
H63	1.0431	0.8005	0.6086	0.036*	
C64	1.0682 (3)	0.6878 (3)	0.6149 (3)	0.0361 (10)	
H64	1.1025	0.6865	0.6686	0.043*	
C65	1.0540 (3)	0.6209 (3)	0.5729 (3)	0.0382 (11)	
H65	1.0779	0.5738	0.5977	0.046*	
C66	1.0051 (4)	0.6234 (3)	0.4947 (3)	0.0426 (12)	
H66	0.9959	0.5780	0.4655	0.051*	
C67	0.9694 (4)	0.6919 (2)	0.4589 (3)	0.0354 (10)	
H67	0.9355	0.6931	0.4052	0.042*	

### Atomic displacement parameters (Å^2^)

**Table:**

	*U*^11^	*U*^22^	*U*^33^	*U*^12^	*U*^13^	*U*^23^
Br1	0.0507 (3)	0.0241 (2)	0.0290 (2)	−0.00541 (19)	0.0163 (2)	0.00051 (17)
Br2	0.0458 (3)	0.0455 (3)	0.0442 (3)	−0.0152 (2)	0.0254 (2)	0.0000 (2)
O1	0.0412 (19)	0.0322 (16)	0.0332 (18)	0.0032 (15)	0.0150 (16)	0.0008 (14)
C1	0.114 (7)	0.103 (7)	0.193 (11)	0.038 (6)	0.073 (8)	0.032 (7)
Cl1	0.0854 (14)	0.0902 (15)	0.167 (2)	−0.0199 (12)	0.0125 (15)	0.0345 (15)
Cl2	0.288 (4)	0.0771 (16)	0.247 (4)	−0.066 (2)	0.181 (4)	−0.0282 (19)
N1	0.0251 (18)	0.0179 (16)	0.0216 (17)	−0.0018 (13)	0.0093 (14)	−0.0006 (13)
O11	0.0392 (18)	0.0219 (15)	0.0278 (16)	0.0056 (13)	0.0176 (14)	0.0026 (12)
O12	0.0473 (19)	0.0242 (16)	0.0258 (16)	0.0014 (14)	0.0135 (14)	0.0034 (13)
C11	0.022 (2)	0.022 (2)	0.027 (2)	−0.0053 (16)	0.0099 (17)	−0.0046 (16)
C12	0.032 (2)	0.023 (2)	0.036 (2)	0.0015 (18)	0.017 (2)	−0.0020 (18)
C13	0.032 (2)	0.027 (2)	0.038 (3)	−0.0015 (18)	0.019 (2)	−0.0086 (18)
C14	0.029 (2)	0.030 (2)	0.029 (2)	−0.0027 (18)	0.0157 (19)	−0.0062 (17)
C15	0.026 (2)	0.025 (2)	0.027 (2)	0.0001 (17)	0.0113 (18)	−0.0008 (16)
C16	0.019 (2)	0.0214 (19)	0.024 (2)	−0.0038 (16)	0.0080 (17)	−0.0052 (16)
C17	0.022 (2)	0.0212 (19)	0.022 (2)	−0.0054 (16)	0.0082 (17)	−0.0046 (15)
C18	0.022 (2)	0.023 (2)	0.021 (2)	−0.0008 (16)	0.0076 (17)	−0.0001 (16)
C19	0.055 (3)	0.048 (3)	0.034 (3)	0.007 (2)	0.026 (2)	−0.005 (2)
C21	0.026 (2)	0.030 (2)	0.027 (2)	0.0039 (18)	0.0117 (18)	−0.0022 (18)
C22	0.032 (2)	0.038 (2)	0.026 (2)	0.0091 (19)	0.0136 (19)	0.0063 (18)
C23	0.027 (2)	0.051 (3)	0.025 (2)	0.011 (2)	0.0121 (19)	0.002 (2)
C24	0.029 (2)	0.044 (3)	0.029 (2)	0.008 (2)	0.0126 (19)	−0.009 (2)
C25	0.029 (2)	0.030 (2)	0.031 (2)	0.0055 (18)	0.0134 (19)	−0.0038 (18)
C26	0.026 (2)	0.026 (2)	0.026 (2)	0.0024 (17)	0.0121 (18)	−0.0019 (17)
C27	0.023 (2)	0.023 (2)	0.026 (2)	−0.0031 (16)	0.0100 (17)	−0.0046 (16)
C28	0.026 (2)	0.024 (2)	0.027 (2)	−0.0011 (17)	0.0127 (18)	−0.0051 (16)
C29	0.041 (3)	0.055 (3)	0.036 (3)	0.002 (2)	0.017 (2)	−0.018 (2)
C31	0.022 (2)	0.0211 (19)	0.029 (2)	−0.0041 (16)	0.0111 (17)	−0.0034 (16)
C32	0.026 (2)	0.021 (2)	0.033 (2)	−0.0009 (17)	0.0153 (18)	−0.0013 (17)
C33	0.027 (2)	0.024 (2)	0.033 (2)	−0.0013 (17)	0.0114 (19)	−0.0016 (18)
C34	0.031 (2)	0.032 (2)	0.034 (2)	−0.0006 (19)	0.010 (2)	0.0037 (19)
C35	0.038 (3)	0.023 (2)	0.051 (3)	0.0031 (19)	0.021 (2)	0.008 (2)
C36	0.053 (3)	0.022 (2)	0.051 (3)	0.002 (2)	0.029 (3)	−0.003 (2)
C37	0.049 (3)	0.025 (2)	0.035 (2)	0.004 (2)	0.022 (2)	0.0009 (18)
N2	0.0253 (18)	0.0185 (16)	0.0202 (16)	0.0015 (13)	0.0110 (14)	0.0017 (13)
O21	0.0441 (19)	0.0212 (15)	0.0266 (16)	0.0079 (14)	0.0184 (14)	0.0019 (12)
O22	0.052 (2)	0.0236 (16)	0.0258 (16)	−0.0005 (14)	0.0197 (15)	0.0030 (12)
C41	0.025 (2)	0.023 (2)	0.024 (2)	−0.0036 (16)	0.0113 (17)	−0.0024 (16)
C42	0.038 (3)	0.022 (2)	0.037 (2)	0.0015 (18)	0.020 (2)	−0.0030 (18)
C43	0.036 (3)	0.030 (2)	0.034 (2)	−0.0006 (19)	0.020 (2)	−0.0113 (19)
C44	0.027 (2)	0.034 (2)	0.028 (2)	−0.0028 (18)	0.0146 (19)	−0.0075 (18)
C45	0.024 (2)	0.031 (2)	0.024 (2)	0.0012 (17)	0.0095 (18)	−0.0019 (17)
C46	0.018 (2)	0.024 (2)	0.025 (2)	−0.0040 (16)	0.0108 (16)	−0.0068 (16)
C47	0.019 (2)	0.0225 (19)	0.023 (2)	−0.0038 (15)	0.0109 (16)	−0.0039 (16)
C48	0.027 (2)	0.024 (2)	0.020 (2)	−0.0006 (17)	0.0118 (17)	0.0008 (16)
C49	0.042 (3)	0.047 (3)	0.028 (2)	0.006 (2)	0.017 (2)	−0.008 (2)
C51	0.028 (2)	0.034 (2)	0.021 (2)	0.0101 (18)	0.0090 (18)	0.0021 (17)
C52	0.031 (2)	0.046 (3)	0.025 (2)	0.012 (2)	0.0132 (19)	0.0081 (19)
C53	0.029 (2)	0.061 (3)	0.018 (2)	0.017 (2)	0.0095 (19)	0.002 (2)
C54	0.026 (2)	0.053 (3)	0.022 (2)	0.018 (2)	0.0037 (18)	−0.010 (2)
C55	0.023 (2)	0.032 (2)	0.024 (2)	0.0090 (17)	0.0070 (17)	−0.0031 (17)
C56	0.023 (2)	0.029 (2)	0.023 (2)	0.0071 (17)	0.0099 (17)	0.0015 (16)
C57	0.022 (2)	0.022 (2)	0.023 (2)	−0.0032 (16)	0.0088 (17)	−0.0069 (16)
C58	0.025 (2)	0.0194 (19)	0.024 (2)	0.0002 (16)	0.0122 (17)	−0.0030 (15)
C59	0.040 (3)	0.061 (3)	0.035 (3)	0.012 (2)	0.008 (2)	−0.020 (2)
C61	0.021 (2)	0.0215 (19)	0.027 (2)	−0.0035 (16)	0.0103 (17)	0.0003 (16)
C62	0.031 (2)	0.022 (2)	0.030 (2)	0.0006 (17)	0.0185 (19)	0.0021 (17)
C63	0.034 (2)	0.028 (2)	0.029 (2)	−0.0018 (19)	0.015 (2)	0.0016 (18)
C64	0.040 (3)	0.039 (3)	0.031 (2)	0.001 (2)	0.015 (2)	0.008 (2)
C65	0.042 (3)	0.027 (2)	0.052 (3)	0.006 (2)	0.024 (2)	0.014 (2)
C66	0.062 (3)	0.023 (2)	0.049 (3)	0.001 (2)	0.028 (3)	−0.003 (2)
C67	0.049 (3)	0.024 (2)	0.037 (3)	0.001 (2)	0.020 (2)	0.0022 (18)

### Geometric parameters (Å, º)

**Table:**

O1—H7	0.80 (2)	C36—C37	1.384 (6)
O1—H8	0.79 (2)	C36—H36	0.9500
C1—Cl2	1.681 (10)	C37—H37	0.9500
C1—Cl1	1.752 (10)	N2—C57	1.357 (5)
C1—H1A	0.9900	N2—C47	1.362 (5)
C1—H1B	0.9900	N2—H2	0.8800
N1—C27	1.352 (5)	O21—C41	1.369 (5)
N1—C17	1.359 (5)	O21—H5	0.79 (2)
N1—H1	0.8800	O22—C51	1.361 (5)
O11—C11	1.355 (5)	O22—H6	0.79 (2)
O11—H3	0.79 (2)	C41—C42	1.390 (5)
O12—C21	1.366 (5)	C41—C46	1.404 (5)
O12—H4	0.79 (2)	C42—C43	1.378 (6)
C11—C12	1.386 (5)	C42—H42	0.9500
C11—C16	1.409 (5)	C43—C44	1.389 (6)
C12—C13	1.381 (6)	C43—H43	0.9500
C12—H12	0.9500	C44—C45	1.385 (5)
C13—C14	1.386 (6)	C44—C49	1.508 (6)
C13—H13	0.9500	C45—C46	1.405 (5)
C14—C15	1.389 (5)	C45—H45	0.9500
C14—C19	1.514 (6)	C46—C47	1.481 (5)
C15—C16	1.405 (5)	C47—C48	1.388 (5)
C15—H15	0.9500	C48—C61	1.399 (5)
C16—C17	1.481 (5)	C48—H48	0.9500
C17—C18	1.390 (5)	C49—H49A	0.9800
C18—C31	1.400 (5)	C49—H49B	0.9800
C18—H18	0.9500	C49—H49C	0.9800
C19—H19A	0.9800	C51—C52	1.399 (6)
C19—H19B	0.9800	C51—C56	1.408 (6)
C19—H19C	0.9800	C52—C53	1.385 (6)
C21—C22	1.399 (6)	C52—H52	0.9500
C21—C26	1.407 (6)	C53—C54	1.392 (7)
C22—C23	1.377 (6)	C53—H53	0.9500
C22—H22	0.9500	C54—C55	1.392 (6)
C23—C24	1.390 (6)	C54—C59	1.512 (6)
C23—H23	0.9500	C55—C56	1.399 (6)
C24—C25	1.394 (6)	C55—H55	0.9500
C24—C29	1.516 (6)	C56—C57	1.474 (5)
C25—C26	1.399 (6)	C57—C58	1.368 (5)
C25—H25	0.9500	C58—C61	1.400 (5)
C26—C27	1.482 (5)	C58—H58	0.9500
C27—C28	1.370 (6)	C59—H59A	0.9800
C28—C31	1.403 (5)	C59—H59B	0.9800
C28—H28	0.9500	C59—H59C	0.9800
C29—H29A	0.9800	C61—C62	1.479 (5)
C29—H29B	0.9800	C62—C63	1.389 (6)
C29—H29C	0.9800	C62—C67	1.400 (6)
C31—C32	1.487 (5)	C63—C64	1.365 (6)
C32—C33	1.388 (6)	C63—H63	0.9500
C32—C37	1.401 (6)	C64—C65	1.389 (6)
C33—C34	1.372 (6)	C64—H64	0.9500
C33—H33	0.9500	C65—C66	1.382 (7)
C34—C35	1.385 (6)	C65—H65	0.9500
C34—H34	0.9500	C66—C67	1.381 (6)
C35—C36	1.379 (6)	C66—H66	0.9500
C35—H35	0.9500	C67—H67	0.9500
			
H7—O1—H8	116 (6)	C36—C37—C32	120.5 (4)
Cl2—C1—Cl1	114.0 (6)	C36—C37—H37	119.7
Cl2—C1—H1A	108.7	C32—C37—H37	119.7
Cl1—C1—H1A	108.7	C57—N2—C47	124.2 (3)
Cl2—C1—H1B	108.7	C57—N2—H2	117.9
Cl1—C1—H1B	108.7	C47—N2—H2	117.9
H1A—C1—H1B	107.6	C41—O21—H5	113 (4)
C27—N1—C17	124.4 (3)	C51—O22—H6	117 (4)
C27—N1—H1	117.8	O21—C41—C42	119.8 (4)
C17—N1—H1	117.8	O21—C41—C46	119.5 (3)
C11—O11—H3	116 (4)	C42—C41—C46	120.7 (4)
C21—O12—H4	110 (3)	C43—C42—C41	120.2 (4)
O11—C11—C12	120.5 (4)	C43—C42—H42	119.9
O11—C11—C16	119.2 (3)	C41—C42—H42	119.9
C12—C11—C16	120.4 (4)	C42—C43—C44	121.3 (4)
C13—C12—C11	120.7 (4)	C42—C43—H43	119.3
C13—C12—H12	119.7	C44—C43—H43	119.3
C11—C12—H12	119.7	C45—C44—C43	117.7 (4)
C12—C13—C14	120.8 (4)	C45—C44—C49	122.0 (4)
C12—C13—H13	119.6	C43—C44—C49	120.4 (4)
C14—C13—H13	119.6	C44—C45—C46	123.2 (4)
C13—C14—C15	118.3 (4)	C44—C45—H45	118.4
C13—C14—C19	120.6 (4)	C46—C45—H45	118.4
C15—C14—C19	121.1 (4)	C41—C46—C45	116.9 (3)
C14—C15—C16	122.6 (4)	C41—C46—C47	123.4 (3)
C14—C15—H15	118.7	C45—C46—C47	119.7 (3)
C16—C15—H15	118.7	N2—C47—C48	116.9 (3)
C15—C16—C11	117.2 (3)	N2—C47—C46	118.5 (3)
C15—C16—C17	120.2 (3)	C48—C47—C46	124.5 (3)
C11—C16—C17	122.6 (3)	C47—C48—C61	121.4 (3)
N1—C17—C18	116.9 (3)	C47—C48—H48	119.3
N1—C17—C16	118.5 (3)	C61—C48—H48	119.3
C18—C17—C16	124.5 (3)	C44—C49—H49A	109.5
C17—C18—C31	121.3 (4)	C44—C49—H49B	109.5
C17—C18—H18	119.3	H49A—C49—H49B	109.5
C31—C18—H18	119.3	C44—C49—H49C	109.5
C14—C19—H19A	109.5	H49A—C49—H49C	109.5
C14—C19—H19B	109.5	H49B—C49—H49C	109.5
H19A—C19—H19B	109.5	O22—C51—C52	121.9 (4)
C14—C19—H19C	109.5	O22—C51—C56	118.2 (3)
H19A—C19—H19C	109.5	C52—C51—C56	119.9 (4)
H19B—C19—H19C	109.5	C53—C52—C51	119.4 (4)
O12—C21—C22	122.1 (4)	C53—C52—H52	120.3
O12—C21—C26	118.4 (3)	C51—C52—H52	120.3
C22—C21—C26	119.5 (4)	C52—C53—C54	122.4 (4)
C23—C22—C21	120.2 (4)	C52—C53—H53	118.8
C23—C22—H22	119.9	C54—C53—H53	118.8
C21—C22—H22	119.9	C53—C54—C55	117.5 (4)
C22—C23—C24	121.7 (4)	C53—C54—C59	122.0 (4)
C22—C23—H23	119.1	C55—C54—C59	120.6 (5)
C24—C23—H23	119.1	C54—C55—C56	122.2 (4)
C23—C24—C25	118.0 (4)	C54—C55—H55	118.9
C23—C24—C29	122.0 (4)	C56—C55—H55	118.9
C25—C24—C29	120.0 (4)	C55—C56—C51	118.7 (4)
C24—C25—C26	121.8 (4)	C55—C56—C57	118.9 (4)
C24—C25—H25	119.1	C51—C56—C57	122.3 (4)
C26—C25—H25	119.1	N2—C57—C58	118.8 (3)
C25—C26—C21	118.7 (4)	N2—C57—C56	118.9 (3)
C25—C26—C27	118.3 (4)	C58—C57—C56	122.3 (3)
C21—C26—C27	123.0 (3)	C57—C58—C61	120.6 (3)
N1—C27—C28	118.8 (4)	C57—C58—H58	119.7
N1—C27—C26	118.2 (3)	C61—C58—H58	119.7
C28—C27—C26	122.9 (3)	C54—C59—H59A	109.5
C27—C28—C31	120.5 (4)	C54—C59—H59B	109.5
C27—C28—H28	119.8	H59A—C59—H59B	109.5
C31—C28—H28	119.8	C54—C59—H59C	109.5
C24—C29—H29A	109.5	H59A—C59—H59C	109.5
C24—C29—H29B	109.5	H59B—C59—H59C	109.5
H29A—C29—H29B	109.5	C48—C61—C58	118.1 (3)
C24—C29—H29C	109.5	C48—C61—C62	121.0 (3)
H29A—C29—H29C	109.5	C58—C61—C62	120.9 (3)
H29B—C29—H29C	109.5	C63—C62—C67	117.6 (4)
C18—C31—C28	118.0 (4)	C63—C62—C61	122.3 (4)
C18—C31—C32	121.1 (4)	C67—C62—C61	120.1 (4)
C28—C31—C32	120.9 (3)	C64—C63—C62	121.8 (4)
C33—C32—C37	117.7 (4)	C64—C63—H63	119.1
C33—C32—C31	122.3 (4)	C62—C63—H63	119.1
C37—C32—C31	119.9 (4)	C63—C64—C65	120.1 (4)
C34—C33—C32	121.9 (4)	C63—C64—H64	120.0
C34—C33—H33	119.1	C65—C64—H64	120.0
C32—C33—H33	119.1	C66—C65—C64	119.5 (4)
C33—C34—C35	119.8 (4)	C66—C65—H65	120.2
C33—C34—H34	120.1	C64—C65—H65	120.2
C35—C34—H34	120.1	C67—C66—C65	120.1 (4)
C36—C35—C34	119.7 (4)	C67—C66—H66	120.0
C36—C35—H35	120.2	C65—C66—H66	120.0
C34—C35—H35	120.2	C66—C67—C62	121.0 (4)
C35—C36—C37	120.4 (4)	C66—C67—H67	119.5
C35—C36—H36	119.8	C62—C67—H67	119.5
C37—C36—H36	119.8		
			
O11—C11—C12—C13	−177.9 (4)	O21—C41—C42—C43	−178.3 (4)
C16—C11—C12—C13	1.6 (6)	C46—C41—C42—C43	0.1 (6)
C11—C12—C13—C14	0.5 (6)	C41—C42—C43—C44	1.1 (6)
C12—C13—C14—C15	−2.1 (6)	C42—C43—C44—C45	−1.4 (6)
C12—C13—C14—C19	177.1 (4)	C42—C43—C44—C49	177.6 (4)
C13—C14—C15—C16	1.6 (6)	C43—C44—C45—C46	0.4 (6)
C19—C14—C15—C16	−177.6 (4)	C49—C44—C45—C46	−178.6 (4)
C14—C15—C16—C11	0.5 (6)	O21—C41—C46—C45	177.5 (3)
C14—C15—C16—C17	−178.5 (4)	C42—C41—C46—C45	−1.0 (6)
O11—C11—C16—C15	177.4 (3)	O21—C41—C46—C47	−3.9 (6)
C12—C11—C16—C15	−2.1 (5)	C42—C41—C46—C47	177.6 (4)
O11—C11—C16—C17	−3.6 (5)	C44—C45—C46—C41	0.8 (6)
C12—C11—C16—C17	176.9 (4)	C44—C45—C46—C47	−178.0 (4)
C27—N1—C17—C18	0.0 (5)	C57—N2—C47—C48	0.3 (5)
C27—N1—C17—C16	−178.2 (3)	C57—N2—C47—C46	−179.0 (3)
C15—C16—C17—N1	178.3 (3)	C41—C46—C47—N2	3.8 (5)
C11—C16—C17—N1	−0.6 (5)	C45—C46—C47—N2	−177.6 (3)
C15—C16—C17—C18	0.2 (6)	C41—C46—C47—C48	−175.5 (4)
C11—C16—C17—C18	−178.7 (4)	C45—C46—C47—C48	3.1 (6)
N1—C17—C18—C31	0.6 (5)	N2—C47—C48—C61	1.1 (6)
C16—C17—C18—C31	178.7 (4)	C46—C47—C48—C61	−179.6 (4)
O12—C21—C22—C23	−179.2 (4)	O22—C51—C52—C53	−180.0 (4)
C26—C21—C22—C23	2.3 (6)	C56—C51—C52—C53	2.1 (6)
C21—C22—C23—C24	−1.2 (6)	C51—C52—C53—C54	−0.6 (6)
C22—C23—C24—C25	−0.8 (6)	C52—C53—C54—C55	−0.4 (6)
C22—C23—C24—C29	178.1 (4)	C52—C53—C54—C59	−179.3 (4)
C23—C24—C25—C26	1.8 (6)	C53—C54—C55—C56	−0.2 (6)
C29—C24—C25—C26	−177.2 (4)	C59—C54—C55—C56	178.7 (4)
C24—C25—C26—C21	−0.7 (6)	C54—C55—C56—C51	1.7 (6)
C24—C25—C26—C27	179.2 (4)	C54—C55—C56—C57	−173.7 (4)
O12—C21—C26—C25	−179.9 (4)	O22—C51—C56—C55	179.4 (3)
C22—C21—C26—C25	−1.4 (6)	C52—C51—C56—C55	−2.6 (6)
O12—C21—C26—C27	0.2 (6)	O22—C51—C56—C57	−5.4 (6)
C22—C21—C26—C27	178.8 (4)	C52—C51—C56—C57	172.7 (4)
C17—N1—C27—C28	−1.8 (6)	C47—N2—C57—C58	−1.2 (6)
C17—N1—C27—C26	175.9 (3)	C47—N2—C57—C56	−179.9 (3)
C25—C26—C27—N1	−142.9 (4)	C55—C56—C57—N2	−147.1 (4)
C21—C26—C27—N1	36.9 (6)	C51—C56—C57—N2	37.7 (5)
C25—C26—C27—C28	34.7 (6)	C55—C56—C57—C58	34.3 (6)
C21—C26—C27—C28	−145.4 (4)	C51—C56—C57—C58	−141.0 (4)
N1—C27—C28—C31	2.8 (6)	N2—C57—C58—C61	0.7 (6)
C26—C27—C28—C31	−174.8 (4)	C56—C57—C58—C61	179.4 (4)
C17—C18—C31—C28	0.4 (6)	C47—C48—C61—C58	−1.5 (6)
C17—C18—C31—C32	−178.8 (4)	C47—C48—C61—C62	179.3 (4)
C27—C28—C31—C18	−2.2 (6)	C57—C58—C61—C48	0.6 (6)
C27—C28—C31—C32	177.0 (4)	C57—C58—C61—C62	179.8 (4)
C18—C31—C32—C33	−22.9 (6)	C48—C61—C62—C63	−25.3 (6)
C28—C31—C32—C33	157.9 (4)	C58—C61—C62—C63	155.5 (4)
C18—C31—C32—C37	154.1 (4)	C48—C61—C62—C67	154.4 (4)
C28—C31—C32—C37	−25.0 (6)	C58—C61—C62—C67	−24.8 (6)
C37—C32—C33—C34	−0.7 (6)	C67—C62—C63—C64	−0.4 (6)
C31—C32—C33—C34	176.5 (4)	C61—C62—C63—C64	179.3 (4)
C32—C33—C34—C35	0.1 (6)	C62—C63—C64—C65	−0.1 (7)
C33—C34—C35—C36	0.2 (7)	C63—C64—C65—C66	0.7 (7)
C34—C35—C36—C37	0.1 (7)	C64—C65—C66—C67	−0.9 (7)
C35—C36—C37—C32	−0.8 (7)	C65—C66—C67—C62	0.4 (7)
C33—C32—C37—C36	1.0 (6)	C63—C62—C67—C66	0.2 (6)
C31—C32—C37—C36	−176.2 (4)	C61—C62—C67—C66	−179.5 (4)

### Hydrogen-bond geometry (Å, º)

**Table:**

*D*—H···*A*	*D*—H	H···*A*	*D*···*A*	*D*—H···*A*
N1—H1···O11	0.88	1.82	2.547 (4)	138
N2—H2···O21	0.88	1.85	2.575 (4)	138
O11—H3···O1	0.79 (2)	1.80 (3)	2.578 (4)	167 (5)
O12—H4···Br1	0.79 (2)	2.43 (2)	3.219 (3)	175 (5)
O21—H5···Br1^i^	0.79 (2)	2.41 (2)	3.200 (3)	170 (5)
O22—H6···Br2	0.79 (2)	2.35 (2)	3.131 (3)	170 (5)
O1—H7···Br2^ii^	0.80 (2)	2.41 (2)	3.206 (3)	173 (5)
O1—H8···Br1	0.79 (2)	2.61 (3)	3.365 (3)	159 (5)

Symmetry codes: (i) −*x*+1, *y*+1/2, −*z*+1/2; (ii) −*x*+1, *y*−1/2, −*z*+1/2.
