# Supplementary figures and images for: Crystal structure of 2,6-bis­(2-hy­droxy-5-methyl­phen­yl)-4-phenyl­pyridinium bromide di­chloro­methane hemisolvate hemihydrate
Source: Acta Crystallogr E Crystallogr Commun. 2015 Nov 18;71(Pt 12):o953–4. doi: 10.1107/S2056989015021386 (PMC4719919; doi:10.1107/S2056989015021386)

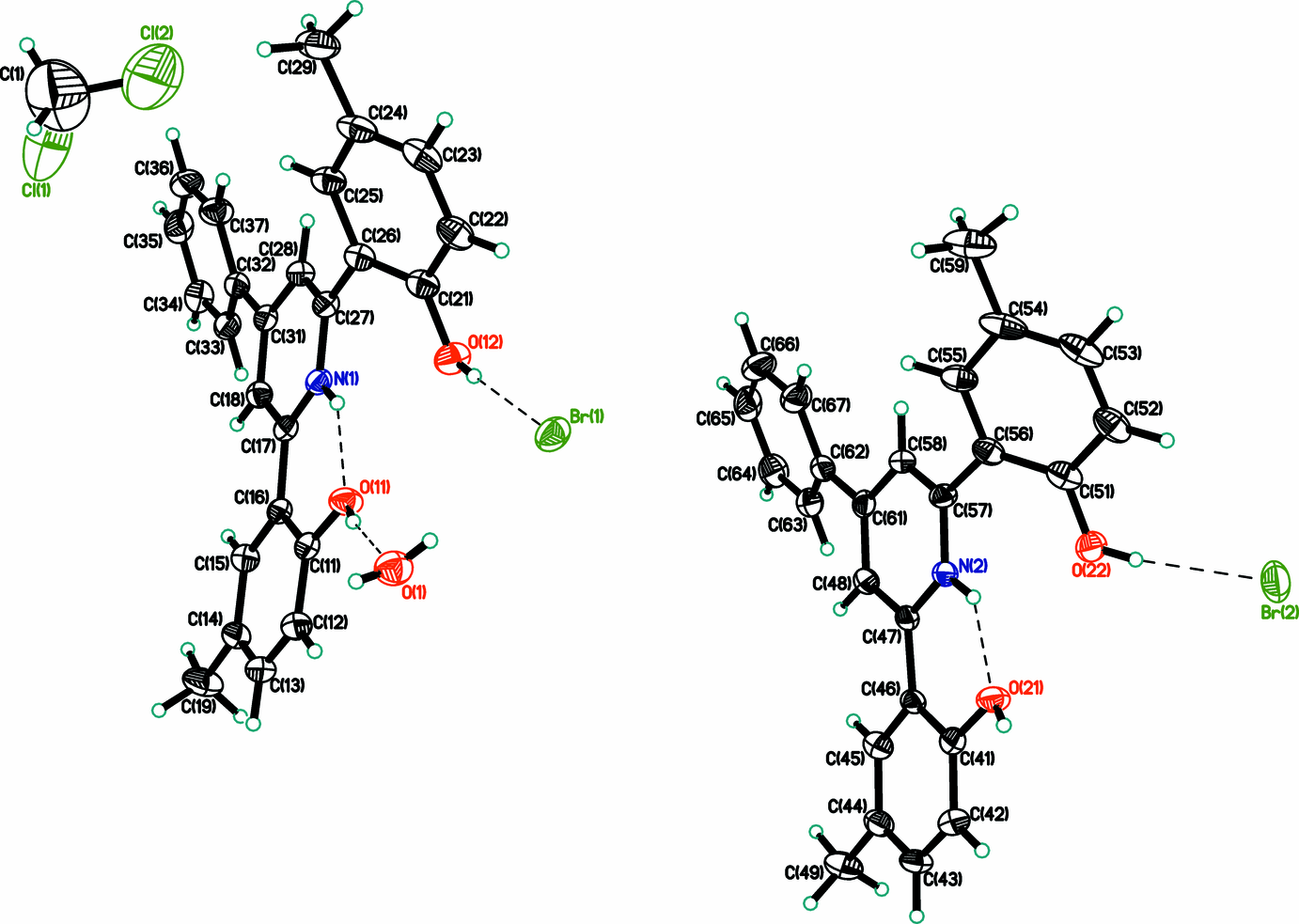

Supplement: Supplementary file 5 [file e-71-0o953-fig1.tif]

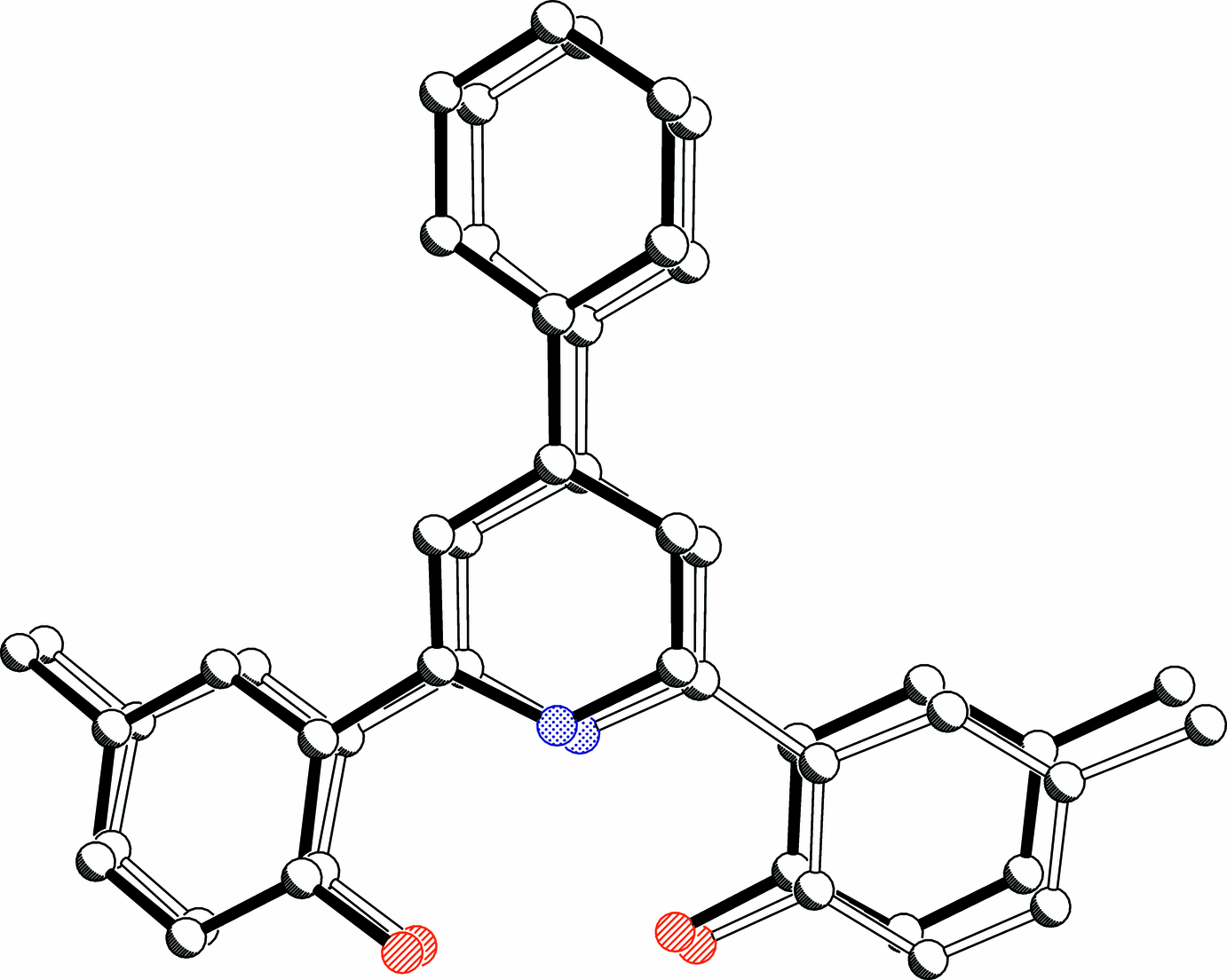

Supplement: Supplementary file 6 [file e-71-0o953-fig2.tif]

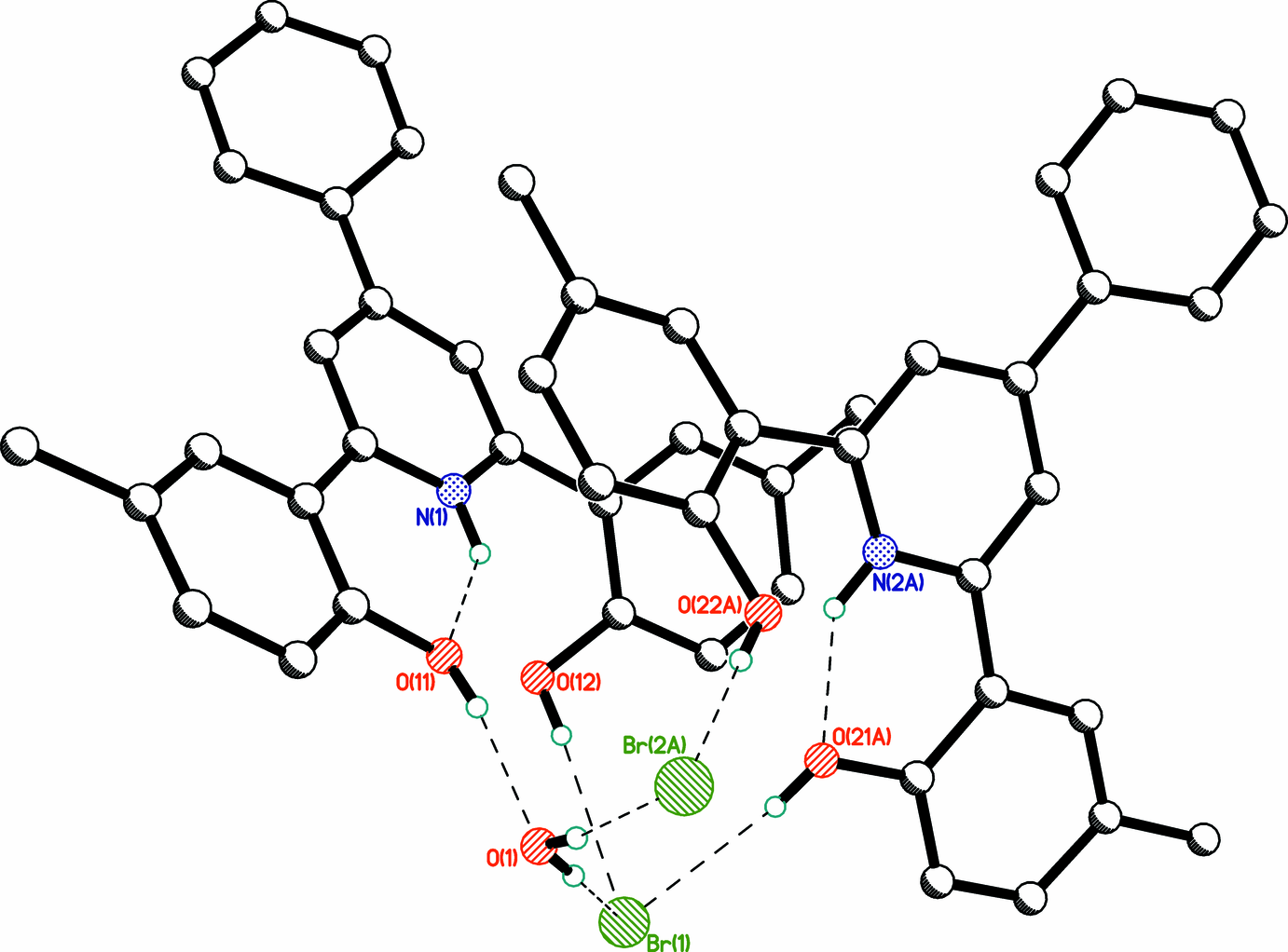

Supplement: Supplementary file 7 [file e-71-0o953-fig3.tif]

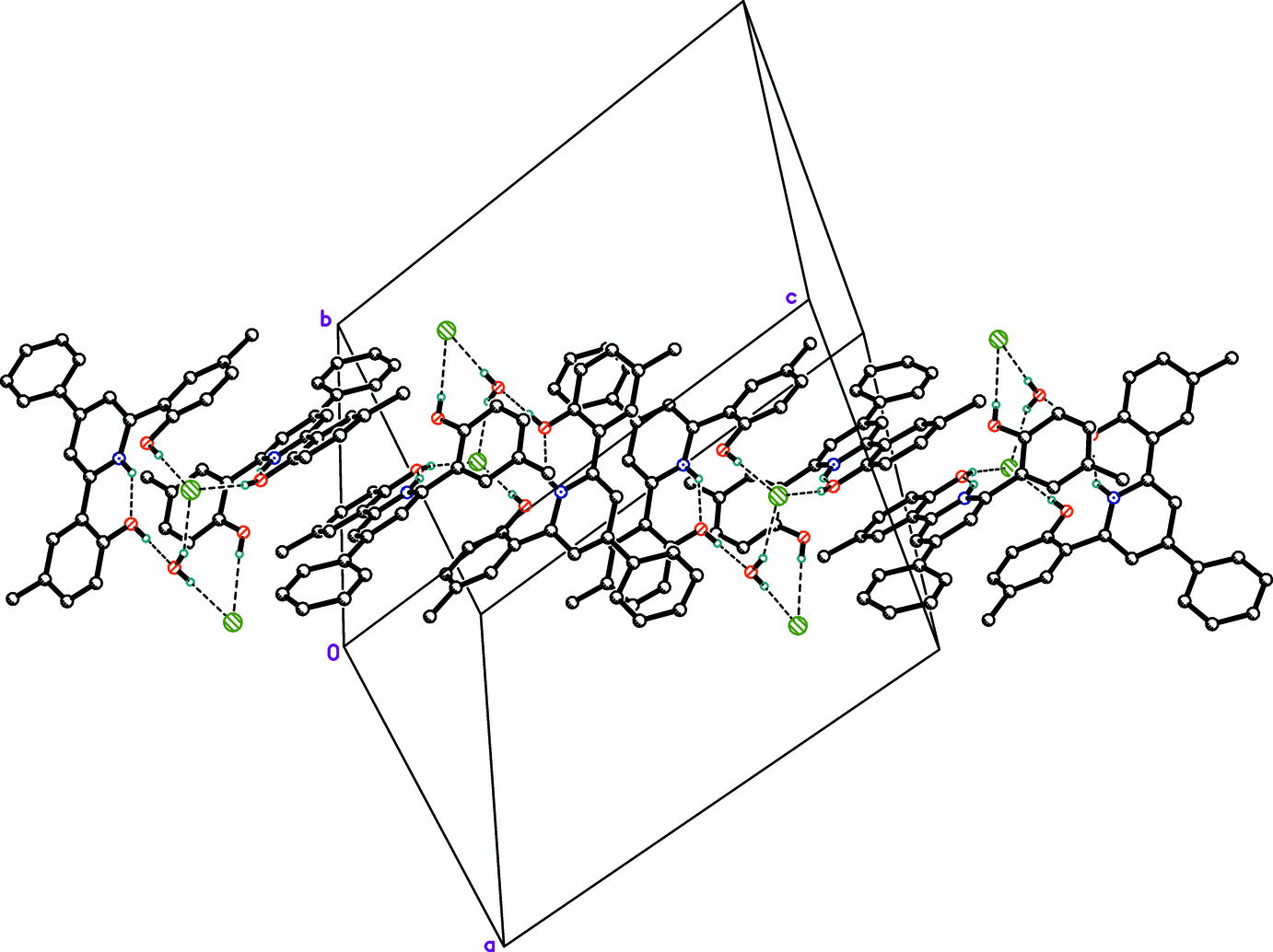

Supplement: Supplementary file 8 [file e-71-0o953-fig4.tif]
